# The Role of Intermittent Fasting in Rheumatoid Arthritis—A Scoping Review

**DOI:** 10.3390/nu18172803

**Published:** 2026-08-27

**Authors:** Martyna Winiarska, Dominika Wiśniewska, Sabina Krupa-Nurcek

**Affiliations:** 1 Faculty of Medicine, Collegium Medicum, University of Rzeszów, 35-310 Rzeszów, Poland; martynawiniarska44@gmail.com (M.W.); dominika.wisniewska.001@gmail.com (D.W.); 2 Department of Surgery, Faculty of Medicine, Collegium Medicum, University of Rzeszów, 35-315 Rzeszów, Poland

**Keywords:** rheumatoid arthritis, intermittent fasting, inflammation, immunomodulation, metabolic health

## Abstract

**Background**: Rheumatoid arthritis (RA) is a chronic autoimmune disease characterized by progressive synovial inflammation, joint destruction, and metabolic abnormalities. In recent years, there has been growing interest in intermittent fasting (IF) as a potential dietary strategy for modulating inflammatory and immunometabolic processes in RA. Preliminary data suggest that IF may lead to reduced joint pain, lower inflammatory markers, improved well-being, and weight loss, but the available evidence is scattered and heterogeneous. The aim of this scoping review was to provide a comprehensive overview of the current state of knowledge regarding the effects of IF on inflammatory, immunological, and metabolic processes in patients with RA. **Methods:** The review was conducted in accordance with the Joanna Briggs Institute methodology and the PRISMA-ScR guidelines. The databases PubMed, Scopus, Web of Science, EBSCO, the Cochrane Library, and Google Scholar were searched (16 March to 12 April 2026) using the Population–Concept–Context model. Full-text observational and experimental studies, as well as reviews, on intermittent fasting in RA were included in the analysis. **Results:** Of the 137 publications identified, 9 studies were included in the review, covering populations from Iran, Luxembourg, Tunisia, Mexico, China, and Turkey. The results indicate that IF may lead to improvements in disease activity, quality of life, and selected metabolic parameters. Several studies reported a beneficial effect of IF on markers of oxidative stress and selected pro-inflammatory cytokines, although other analyses did not confirm significant changes in inflammatory biomarkers. Preclinical data have demonstrated strong anti-inflammatory and immunoregulatory effects of IF, including a reduction in pro-inflammatory cytokines and an increase in the Treg population. The heterogeneity of IF protocols, population differences, and small sample sizes limit the ability to interpret the results unequivocally. **Conclusions:** The available evidence suggests that intermittent fasting may represent a promising, nonpharmacological strategy to support the treatment of RA, particularly in terms of improving immunometabolic parameters and subjective disease symptoms. However, due to methodological limitations and the lack of long-term studies, further well-designed randomized clinical trials are needed to evaluate the efficacy and safety of IF in this population.

## 1. Introduction

Rheumatoid arthritis (RA) is a chronic, systemic autoimmune disease characterized by progressive synovial inflammation, joint destruction, and an increased risk of cardiovascular and metabolic complications [1,2]. Despite significant therapeutic advances, including disease-modifying antirheumatic drugs (DMARDs) and biologic therapies, some patients continue to experience active inflammation, fatigue, metabolic disorders, and a reduced quality of life [3]. In recent years, there has been growing interest in the role of dietary interventions as a complement to pharmacological treatment, particularly intermittent fasting (IF), which may modulate the immune response and inflammatory processes [3,4].

Intermittent fasting encompasses various regimens of temporary food restriction, such as time-restricted eating (TRE), alternate-day fasting (ADF), or periodic fasts lasting several days [5]. The biological mechanisms associated with IF include, among others, a shift from glucose to ketone metabolism, activation of autophagy, reduction in oxidative stress, improvement in insulin sensitivity, and modulation of the gut microbiota [6]. In the context of autoimmune diseases, immunomodulatory effects are of particular importance, such as reduced Th17 lymphocyte activity, increased Treg populations, reduced levels of pro-inflammatory cytokines (IL-6, TNF-α), and improved mitochondrial function in immune system cells [5,7].

In RA, metabolic disorders, chronic inflammation, and increased oxidative stress are observed, making this population potentially receptive to the benefits of dietary interventions aimed at improving immunometabolic homeostasis [8]. Preliminary clinical studies suggest that intermittent fasting in RA may lead to reduced joint pain, lower inflammatory markers, improved well-being, and weight loss, although the available evidence remains limited and heterogeneous [9,10,11,12,13]. Importantly, IF may influence the gut–immune axis, whose dysfunction is considered one of the key elements in the pathogenesis of RA [14].

**Figure 1:**
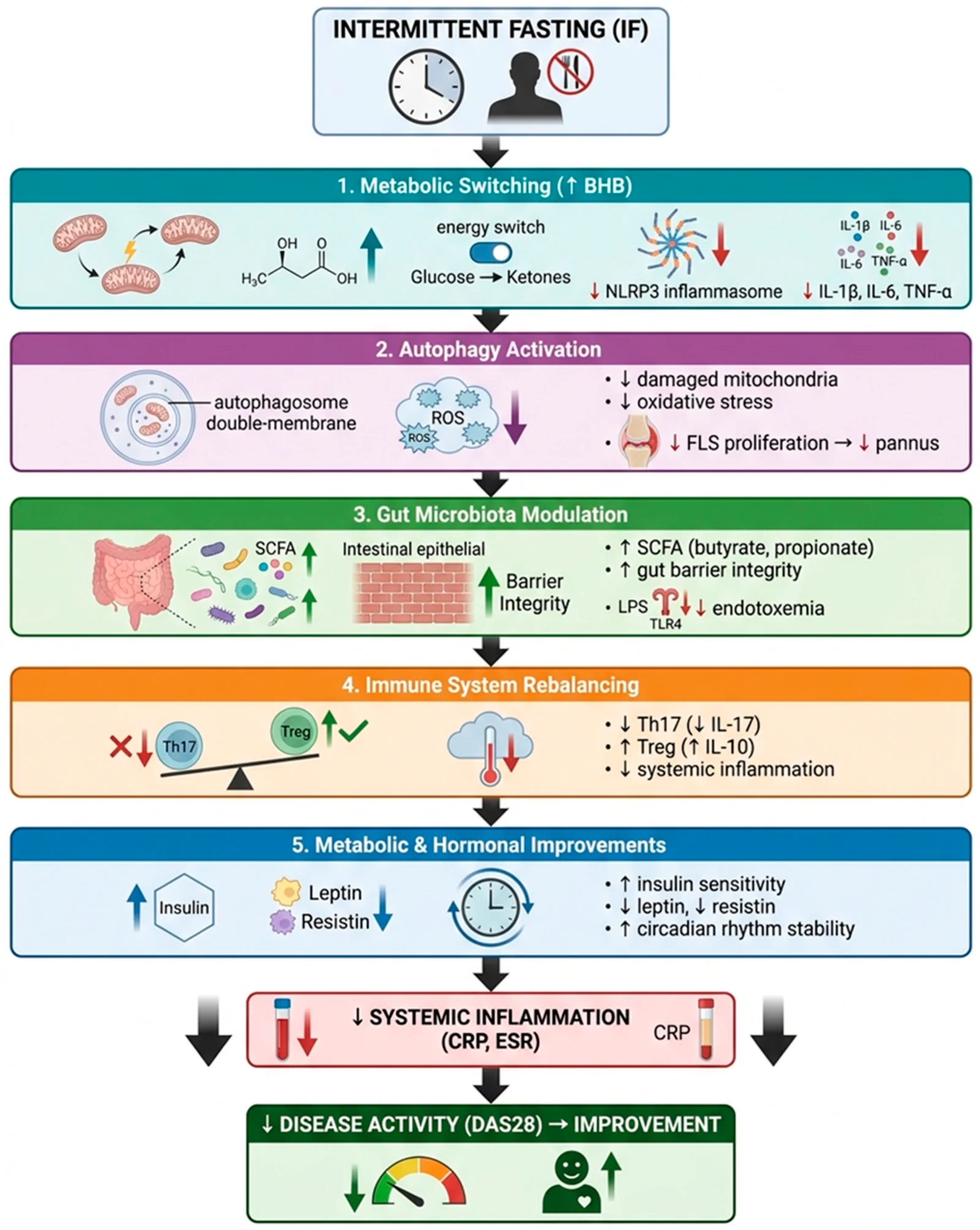
The mechanism of the effect of intermittent fasting on rheumatoid arthritis.

Despite a growing number of reports, there is a lack of comprehensive reviews synthesizing the current state of knowledge on the role of intermittent fasting in RA, covering both biological mechanisms and clinical trial results [7,15]. Understanding the potential benefits, limitations, and safety of IF in this population is crucial for assessing whether this intervention can be a valuable component of a therapeutic strategy [16]. The aim of this review is to present the current evidence regarding the effects of intermittent fasting on inflammatory, immunological, and metabolic processes in RA and to identify areas requiring further research. Figure 1 illustrates the mechanism by which intermittent fasting affects RA.

## 2. Materials and Methods

### 2.1. Study Design

This scoping review was conducted in accordance with the Joanna Briggs Institute (JBI) methodology and presented in a manner consistent with the PRISMA-ScR guidelines. The use of this review format allowed for a comprehensive depiction of the scope, characteristics, and properties of the available data regarding the role of intermittent fasting in rheumatoid arthritis, particularly given the significant diversity of research designs, endpoints assessed, and methodological quality in this field. In accordance with JBI recommendations, no formal assessment of risk of bias was conducted, as the purpose of a scoping review is to identify and synthesize existing evidence, rather than to evaluate the effectiveness of an intervention. A protocol covering the research question, PCC framework, inclusion criteria, search strategy, and data extraction procedures was prepared prior to the start of the study, although it was not registered in an external database [17].

The review was conducted in accordance with the methodology described in the literature by the Joanna Briggs Institute and based on the guidelines for reporting systematic reviews and meta-analyses in the version intended for scoping reviews (PRISMA-ScR) [18,19].

### 2.2. Inclusion and Exclusion Criteria

As part of this review, a research question was formulated that precisely defined the population, concept, and context of the analyses, which made it possible to assess the current state of knowledge regarding the use of intermittent fasting in rheumatoid arthritis. To enhance transparency and avoid repetition, the inclusion criteria were further organized according to the PICOS framework, which allowed for a clear presentation of the population, interventions, comparisons, outcomes, and study types included in the scoping review.

Population The population of interest comprised individuals diagnosed with rheumatoid arthritis, as well as animal models of rheumatoid arthritis used in preclinical studies. No restrictions were applied regarding sex, age, disease duration, or disease severity.

Concept The concept of interest was intermittent fasting and dietary interventions based on periodic energy restriction, including time-restricted eating, the 5:2 protocol, alternate-day fasting, and other forms of cyclic calorie restriction.

Context The review considered clinical and preclinical settings in which intermittent fasting was investigated in relation to rheumatoid arthritis, including its potential effects on disease activity, inflammatory and immunological processes, metabolic parameters, oxidative stress, and quality of life.

Types of studies The analysis included observational studies, experimental clinical trials, original preclinical studies in animal models, and systematic and narrative reviews, provided they offered relevant data on this topic. The definition of the scope of the research projects was consistently maintained in the abstract, methods, and results sections to ensure full methodological transparency.

### 2.3. Search Strategy

As part of the review, a literature search was conducted in the following databases: PubMed, Scopus, EBSCO, Web of Science, Google Scholar, and the Cochrane Library. The search strategy used keywords related to intermittent fasting and rheumatoid arthritis, such as “intermittent fasting,” “fasting,” “time-restricted eating,” “periodic calorie restriction,” “rheumatoid arthritis,” and “RA,” as well as various combinations of these terms linked by the operators AND and OR. All identified publications underwent an initial screening based on titles and abstracts to exclude studies unrelated to the review’s topic. Any discrepancies in the assessment of article eligibility were resolved through joint discussion among the researchers until full consensus was reached. The database search was conducted between 16 March 2026 and 12 April 2026, while the literature selection and data extraction process continued until 10 June 2026.

The selection of databases was based on JBI recommendations for comprehensive mapping of available evidence and on the interdisciplinary nature of the topic, encompassing dietetics, immunology, rheumatology, and lifestyle medicine. PubMed, Scopus, and Web of Science were included as key biomedical and multidisciplinary databases, providing broad access to clinical and epidemiological studies. EBSCO databases (Medline Complete, CINAHL) were included to obtain additional clinical, nursing, and health sciences literature. The Cochrane Library was used to identify existing systematic reviews and evidence of interventions. Google Scholar served as a supplementary source, enabling access to gray literature and publications not included in traditional databases. This combination maximizes search sensitivity and minimizes the risk of omitting relevant data.

The search strategy was developed in accordance with the PRISMA-ScR guidelines, the JBI Manual for Evidence Synthesis, and the principles of transparency in the search process. The search results are presented in Table 1.

Although the PRISMA flowchart presents two thematic pathways, for this review on intermittent fasting in RA, the literature search was conducted as a single, coherent search encompassing all terms related to periodic energy restriction and rheumatoid arthritis. The division into thematic categories occurred only during the data selection and extraction phase, reflecting the structure of the research question rather than two separate search processes.

### 2.4. Extraction of Data

A form developed in accordance with the JBI guidelines for scoping reviews [20] was used to compile the data; it included key information extracted from the analyzed publications. The data extraction process—referred to in scoping reviews as “data charting” [19,20]—was conducted independently by two reviewers. The Population–Concept–Context (PCC) framework was used to identify relevant studies. Information such as the first author’s last name, year of publication, country, main results, and key conclusions was extracted from each article. The entire data compilation process was carried out by the authors using Microsoft Excel.

### 2.5. Process for Including Publications in the Review

**Figure 2:**
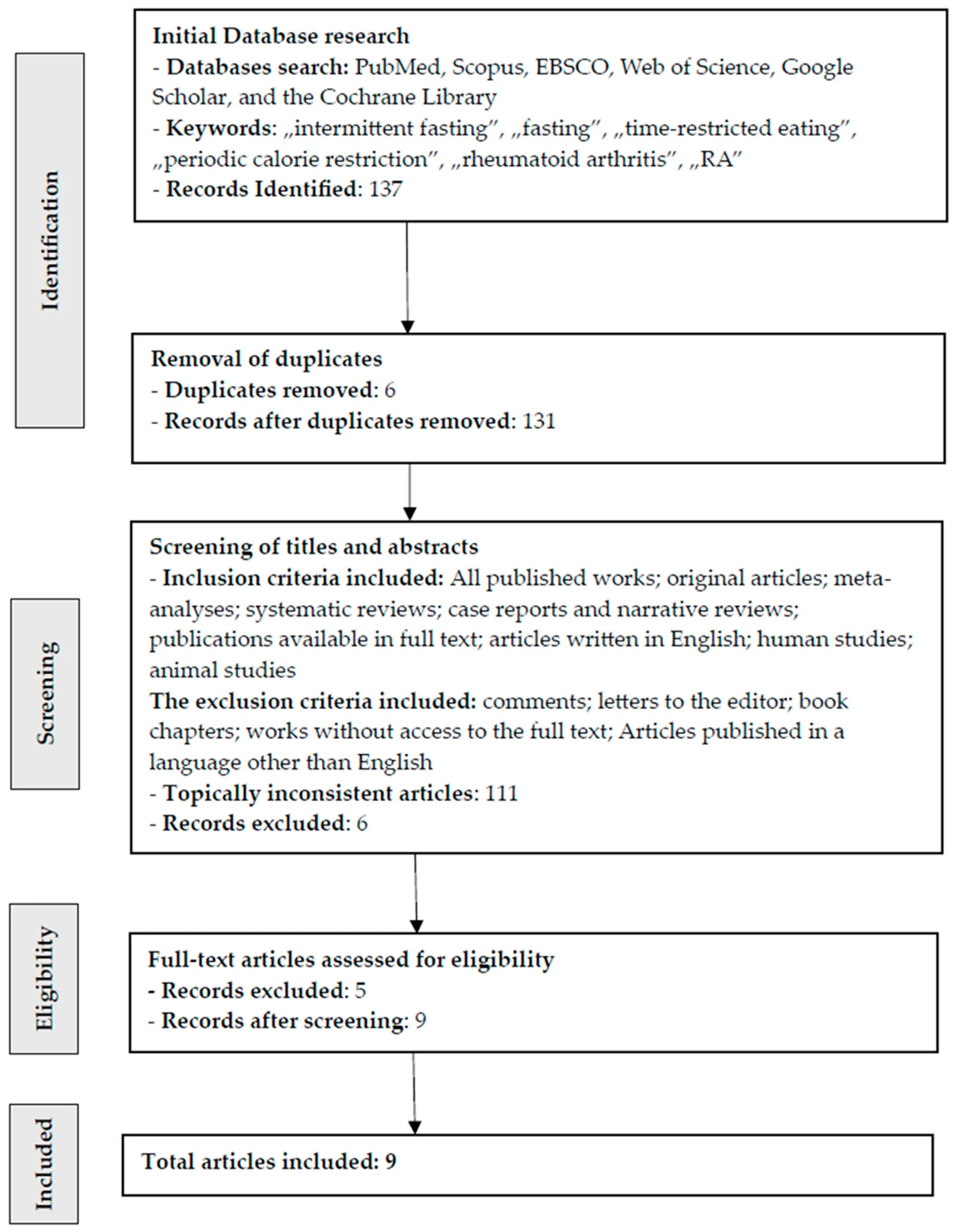
Literature search and selection flowchart for this review.

As part of our scoping review, 137 publications were initially identified, of which 9 ultimately met the inclusion criteria and were analyzed. The selection process was conducted with the participation of three reviewers. Two of them (D.W. and M.W.) independently assessed the titles and abstracts and then independently analyzed the full texts of articles potentially eligible for inclusion. Any discrepancies at any stage were resolved through discussion. In cases of disagreement, the third reviewer (S.KN) acted as an arbitrator. This approach ensured the independence of the evaluation and reduced the risk of selection bias, in accordance with the recommendations of the JBI and PRISMA-ScR. All of the studies analyzed were closely related to the topic of the review (Figure 1). After removing duplicates (*n* = 6), 131 records remained. Subsequently, after applying the inclusion and exclusion criteria (*n* = 6) and rejecting articles that were thematically inconsistent (*n* = 111), 14 publications were selected for further evaluation. Of these, 5 did not include the full text and were excluded. Ultimately, after meeting all methodological requirements, 9 articles were included in the review. The studies originated from the following countries: Iran (*n* = 3), Luxembourg (*n* = 2), Tunisia (*n* = 1), Mexico (*n* = 1), China (*n* = 1), and Turkey (*n* = 1). The results are summarized in Table 2 and Figure 2.

The process of study selection followed PRISMA-ScR recommendations and was conducted using predefined eligibility criteria. Titles and abstracts were independently screened by two reviewers, followed by independent full-text assessment. Discrepancies were resolved through discussion, and a third reviewer acted as an arbitrator when needed, which minimized selection bias. A structured JBI-based data charting form was used to ensure consistency in data extraction. The detailed numerical flow of records is presented in Figure 2, while the search strategy is summarized in Table 1; therefore, these elements are not repeated in the text to avoid duplication.

The “Overall assessment” column summarizes the direction of findings (positive, negative, or inconclusive) and indicates areas requiring further research. In line with the limitations described in the Introduction, additional studies are needed to evaluate long-term effects of IF, its impact on inflammatory and oxidative stress biomarkers, differences between fasting protocols, population-specific responses, and immunometabolic mechanisms that remain insufficiently explored in clinical settings.

### 2.6. Justification for the Lack of a Formal Assessment of Methodological Quality

Given the wide variety of studies included in this review—which differ significantly in terms of study design, population characteristics, interventions, and outcome measures—the use of a single, universal tool to assess the risk of bias would be both impossible and methodologically unjustified. At the same time, the methodological limitations of the available publications—including small sample sizes, the observational nature of the analyses, and subjective methods of symptom assessment—were taken into account when interpreting the results and are discussed in detail in the Section 6. The review protocol was not registered or made available in an external repository. All stages of the procedure—including database selection, development of the search strategy, the study selection process, and data extraction—were conducted systematically to ensure transparency and the reproducibility of the procedures.

## 3. Key Aspects of Intermittent Fasting in RA

Intermittent fasting is one of the most promising nonpharmacological strategies to support the treatment of rheumatoid arthritis, and its effects include both the modulation of inflammatory processes and improvements in metabolic parameters [2,9]. Available studies—despite significant heterogeneity—point to consistent patterns of effects, including reduced disease activity, improved quality of life, and beneficial changes in immunometabolic markers. An analysis of the collected evidence allows us to identify common mechanistic and clinical elements that form the foundation for further evaluation of the role of intermittent fasting in the treatment of RA [14,16].

### 3.1. IF as an Intervention Affecting the Inflammatory Process

IF is increasingly being studied as a potential intervention for modulating the inflammatory process in RA. Data from clinical trials, observational studies, reviews, and preclinical analyses indicate that IF may influence key aspects of the disease’s pathophysiology, including pro-inflammatory cytokine activity, oxidative stress, immune cell function, and metabolic regulation [24,25,26]. As emphasized in the document, intermittent fasting may lead to reduced joint pain, lower inflammatory markers, improved well-being, and weight loss, although the evidence remains heterogeneous [27]. Several clinical trials have shown that IF can lead to a reduction in disease activity, suggesting its potential impact on the inflammatory process. A retrospective analysis by Ben Nessib et al. showed that intermittent fasting may result in a rapid improvement in rheumatoid arthritis activity, and this effect persisted for up to three months [9]. These findings are supported by a meta-analysis by Liu et al., which concluded that IF has a positive effect on disease activity, as indicated by both observational studies and RCTs [13]. At the same time, data on the effect of IF on inflammatory markers are mixed. A randomized trial by Tavakoli et al. showed that the 16:8 protocol had a beneficial effect on certain markers of oxidative stress, inflammatory markers, and liver enzymes [11]. In contrast, Ranjbar et al. did not confirm significant changes in inflammatory markers, even though they observed clinical improvement [10]. As noted in the document, IF has no significant effect on inflammation and markers of oxidative stress, which underscores the heterogeneity of the results [27,28].

Preclinical studies provide strong evidence for the immunomodulatory effects of IF. In a mouse model, Cuevas-Martínez et al. demonstrated that IF exerts significant anti-inflammatory and immunoregulatory effects, including a reduction in pro-inflammatory cytokines and improved regulation of the immune response [29]. These results are consistent with the mechanisms described in the introduction to this document, which indicates that IF can decrease Th17 lymphocyte activity, increase the Treg population, and reduce levels of pro-inflammatory cytokines (IL-6, TNF-α). These mechanisms also include improved mitochondrial function, activation of autophagy, and reduction in oxidative stress—processes that are key to the regulation of inflammation in RA. Reviews by Hansen and Gunes-Bayir emphasize that dietary strategies based on energy restriction can “reduce oxidative stress and influence the gut microbiome”, which further supports immune regulation [21,22,23].

**Figure 3:**
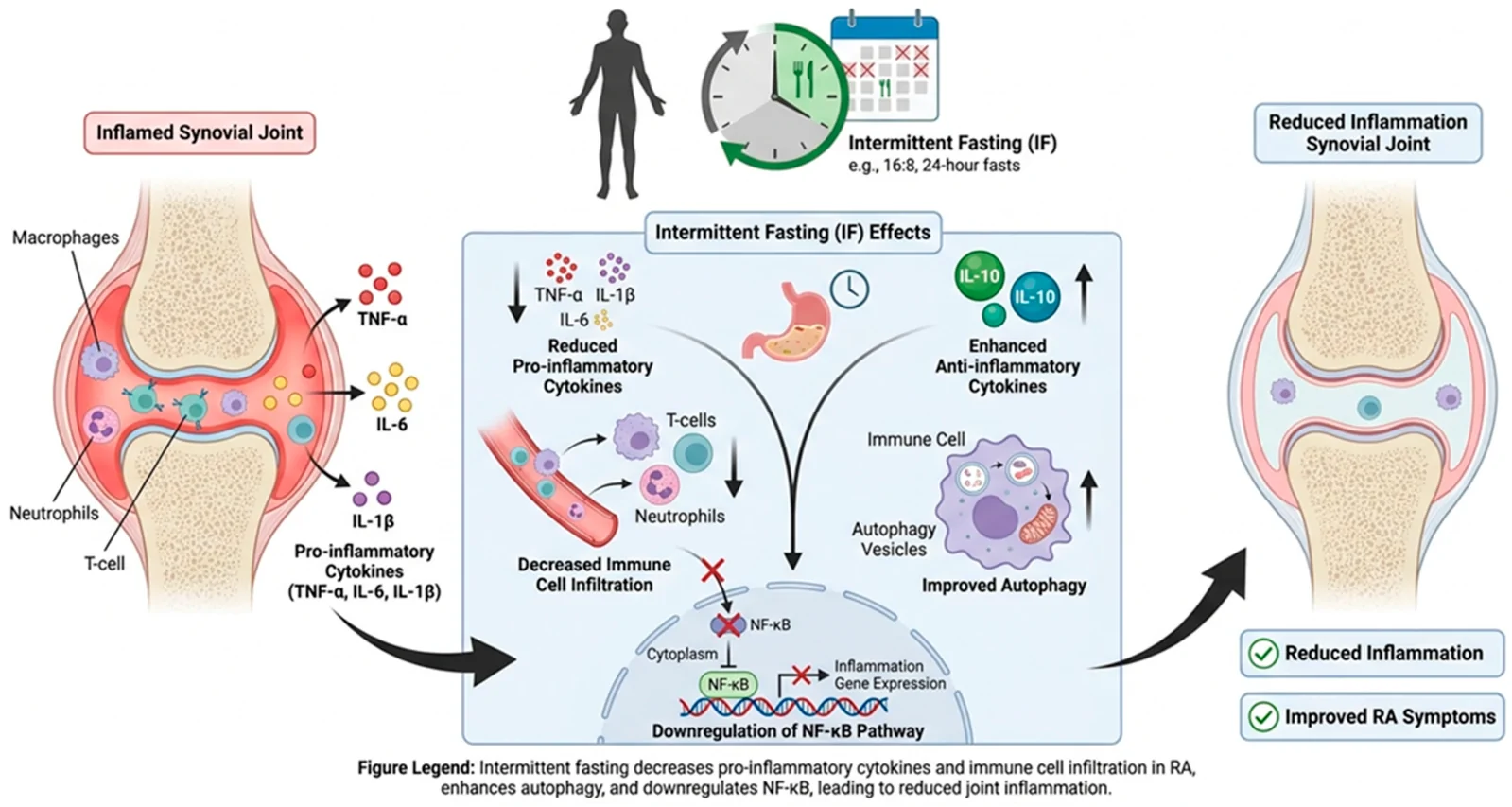
Mechanism of IF reducing inflammation in RA.

Differences in study results may stem from population heterogeneity, IF regimens, and the duration of the interventions. As noted in the document, these discrepancies may stem from differences in study populations (postmenopausal women vs. mixed patient groups), IF regimens (e.g., 16:8 vs. other protocols), intervention duration, and the methods used to assess biomarkers. Studies involving postmenopausal women observed different inflammatory responses compared to mixed populations, suggesting a possible influence of hormonal and metabolic factors [10,11,12]. Narrative reviews also emphasize that IF may act by reducing body weight and improving metabolic parameters, which indirectly influences the inflammatory process. As noted, dietary strategies may support weight management, improve metabolic health, and reduce oxidative stress, which may translate into improved clinical outcomes [30,31]. The collected data indicate that IF may modulate the inflammatory process in RA by influencing pro-inflammatory cytokines, oxidative stress, immune cell function, and metabolic regulation. Results from clinical and observational studies suggest improvements in disease activity and selected inflammatory markers, although not all analyses confirm these effects [13,24]. Preclinical data provide strong support for the biological rationale of IF as an anti-inflammatory intervention. At the same time, the heterogeneity of the studies, small sample sizes, and the variety of IF protocols limit the ability to formulate unambiguous clinical recommendations. As emphasized in the document, study results remain heterogeneous, and a full assessment of the efficacy of IF requires well-designed, multicenter randomized trials. Figure 3 illustrates the mechanism by which IF reduces inflammation in RA.

### 3.2. IF as a Complementary Therapy in RA

IF is increasingly viewed as a promising strategy to support the treatment of RA, particularly in light of its potential impact on inflammatory, metabolic, and immunological processes. The reviewed publications emphasized that IF does not replace standard pharmacological treatment but can serve as a valuable adjunct, supporting immunometabolic regulation and improving patients’ overall well-being. As noted in the document, fasting is a promising adjunctive therapy for RA, although the evidence remains limited and methodologically heterogeneous. Several clinical and observational studies have shown that IF can lead to improved disease activity, suggesting its potential role as an adjunctive intervention. A retrospective analysis by Ben Nessib et al. showed that intermittent fasting practiced during Ramadan may result in a rapid improvement in rheumatoid arthritis activity, and this effect persisted for up to three months [9]. Similar conclusions were presented in a meta-analysis by Liu et al., which found that IF has a positive effect on disease activity, a finding supported by both observational and randomized studies [13].

Although not all studies demonstrated clear benefits in terms of inflammatory markers, some of them reported improvements in clinical symptoms, quality of life, and metabolic parameters. In the study by Ranjbar et al., IF did not significantly affect inflammatory markers; however, the authors highlighted beneficial changes in selected clinical outcomes, suggesting that the effects of IF may extend beyond classic biomarkers of inflammation [12]. The study results suggest that IF may support the treatment of RA by improving metabolic parameters, reducing body weight, and decreasing oxidative stress. Tavakoli et al. demonstrated that the 16:8 protocol led to beneficial changes in markers of oxidative stress, selected inflammatory markers, and liver enzymes in overweight and obese postmenopausal women. The authors emphasized that IF may be an effective nonpharmacological intervention in the treatment of RA, particularly in populations with metabolic disorders [11].

Narrative reviews indicate that dietary interventions based on intermittent energy restriction may support weight regulation, improve metabolic health, and reduce oxidative stress—all of which play a significant role in the pathophysiology of RA [21,23]. The document emphasizes that dietary strategies may contribute to better outcomes in RA, which further strengthens the case for IF as an adjunctive therapy. Preclinical data provide strong evidence for the immunomodulatory effects of IF. In a mouse model, Cuevas-Martínez et al. demonstrated that IF exerts significant anti-inflammatory and immunoregulatory effects, including a reduction in pro-inflammatory cytokines and improved regulation of the immune response [29]. These mechanisms are consistent with those described in the introduction to this document, where it was noted that IF can reduce Th17 lymphocyte activity, increase the Treg population, and improve mitochondrial function in immune cells. Although these data come from preclinical studies, they provide a strong biological rationale for the use of IF as an intervention to support the treatment of RA. Reviews by Hansen emphasize that IF can modulate the gut microbiota, which further influences immune regulation and inflammatory processes [21,22].

**Figure 4:**
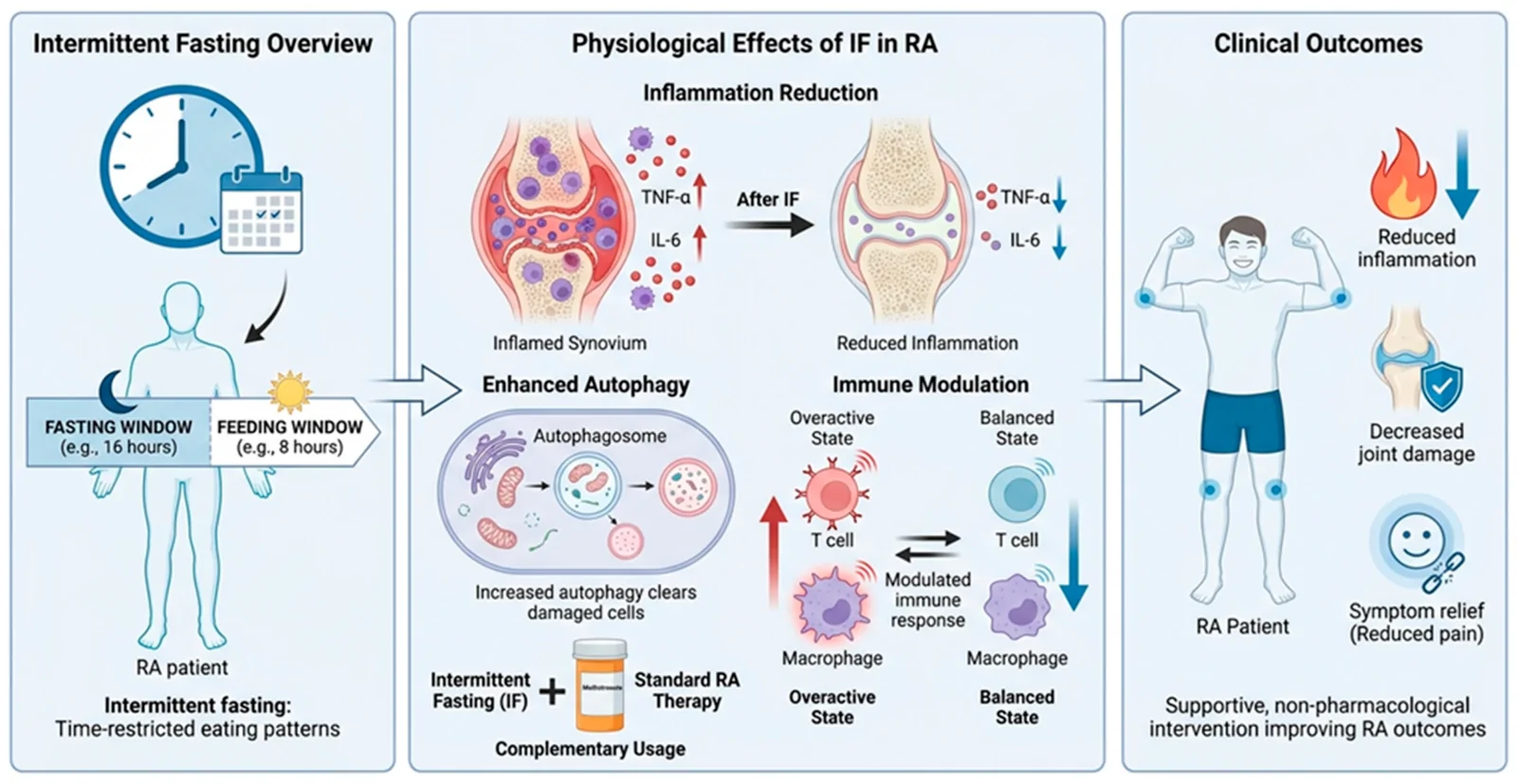
IF as a complementary therapy in RA.

Despite promising results, IF as an adjunctive therapy requires further research with high methodological quality. The publications analyzed repeatedly highlighted limitations such as small sample sizes, short intervention periods, the variety of IF protocols, and the heterogeneity of the study populations. As noted in the document, the literature on fasting in RA is limited and consists of short-term studies, which makes it difficult to formulate clear clinical recommendations. At the same time, the consistency of the observed effects—including improvements in disease activity, metabolic parameters, and subjective symptoms—suggests that IF may be a valuable component of adjunctive therapy, particularly in patients with metabolic disorders or excess body weight. Figure 4 illustrates IF as a complementary therapy in RA.

## 4. Discussion

The results of the studies analyzed indicate that IF may be a promising strategy to support the treatment of RA; however, the available evidence remains limited, methodologically heterogeneous, and often based on small sample sizes. In the clinical trials, observational studies, and systematic reviews included in this review, both beneficial effects of IF and inconclusive results were observed, which requires cautious interpretation.

Several studies have shown that IF may lead to a reduction in disease activity and an improvement in subjective symptoms of RA. A retrospective analysis by Ben Nessib et al. demonstrated rapid improvement in disease activity among patients fasting during Ramadan, persisting for up to three months after the end of the fast [9]. Similar observations were reported in a meta-analysis by Liu et al., where both observational and randomized studies demonstrated the beneficial effect of IF on disease activity [13]. These results are consistent with narrative reviews, which highlight the potential of IF as an adjunctive intervention, particularly in the context of weight loss, improvement in metabolic parameters, and reduction in oxidative stress [21,23]. This points to a certain consistency in the evidence suggesting that IF may influence key pathophysiological mechanisms of RA.

The results regarding the effect of IF on inflammatory markers and oxidative stress are more varied. A randomized study by Tavakoli et al. demonstrated improvements in selected inflammatory markers and liver enzymes in overweight and obese postmenopausal women [11]. However, Ranjbar et al., did not confirm a significant effect of IF on inflammatory markers, although they did note an improvement in certain clinical outcomes [10]. These discrepancies may stem from differences in study populations (postmenopausal women vs. mixed patient groups), IF regimens (e.g., 16:8 vs. other protocols), intervention duration, and the methods used to assess biomarkers. This highlights the need for standardization of IF protocols and longer-term intervention studies.

A study by Cuevas-Martínez et al. using a mouse model confirmed the strong anti-inflammatory and immunomodulatory effects of IF, including a reduction in pro-inflammatory cytokines and improved immune regulation [29]. These results are consistent with the mechanisms described in the literature, including, among others, a reduction in Th17 lymphocyte activity, an increase in the Treg population, and improved mitochondrial function. Although preclinical data support the biological rationale for IF, their translation to the RA patient population requires caution, as animal models do not fully reflect the complexity of the disease in humans.

Reviews by Hansen and Gunes-Bayir emphasize that IF may serve as a valuable adjunctive intervention, particularly in terms of modulating the gut microbiota, reducing oxidative stress, and improving metabolic parameters [21,22,23]. At the same time, the authors point out the limited number of long-term studies and the lack of conclusive data regarding the safety of IF in various patient groups (e.g., the elderly, patients with comorbidities).

The variety of fasting protocols used across the included studies may explain some of the observed discrepancies in clinical and biochemical outcomes. For instance, the 16:8 time-restricted eating (TRE) pattern evaluated in postmenopausal women primarily targets metabolic switching and daily circadian balance, whereas religious daytime fasting (such as during Ramadan) involves altered sleep–wake cycles and fluid restriction during daylight hours. Furthermore, preclinical regimens often involve continuous or longer fasting windows that are difficult to implement in human practice. These biological differences across protocols highlight the need for standardized fasting regimens in future clinical trials.

## 5. Conclusions

The results of available studies indicate that IF may be a promising, nonpharmacological strategy to support the treatment of rheumatoid arthritis. Both observational studies and randomized clinical trials suggest that IF may lead to reduced disease activity, improved quality of life, weight loss, and beneficial changes in selected inflammatory and metabolic markers. Preclinical data further confirm the immunomodulatory potential of IF, including a reduction in pro-inflammatory cytokines and improved regulation of the immune response.

At the same time, study results remain heterogeneous, and some analyses have not demonstrated clear benefits regarding inflammatory markers or oxidative stress. Differences in study populations, IF regimens, intervention duration, and endpoints used limit the ability to formulate unambiguous clinical recommendations. Narrative and systematic reviews highlight the potential of IF but also point to a lack of long-term studies with high methodological quality.

In summary, the available evidence suggests that intermittent fasting may be a valuable adjunct to standard RA treatment, particularly in terms of improving immunometabolic parameters and patients’ overall well-being. However, for IF to be unequivocally incorporated into clinical guidelines, well-designed, multicenter randomized trials with adequate statistical power and a long follow-up period are necessary to assess both the efficacy and safety of this intervention.

## 6. Limitations

The interpretation of the results of this review requires consideration of several important limitations, stemming from both the characteristics of the available studies and the specific nature of the scoping review methodology. First, the included studies were characterized by considerable heterogeneity in study designs (study heterogeneity), encompassing various intermittent fasting regimens, different patient populations, varying intervention durations, and a wide range of endpoints. This diversity limits the possibility of direct comparisons between studies and makes it difficult to draw unambiguous clinical conclusions.

Another limitation is the small sample size in most interventional studies (small samples), which reduces the statistical power of the analyses and increases the risk of Type I and Type II errors. Some observational studies also lacked control groups, making it difficult to assess the cause-and-effect relationship of the observed effects. Furthermore, some of the results were based on subjective measures, such as self-reported symptoms or quality of life, which may be susceptible to the placebo effect or reporting bias.

Another significant limitation is the lack of long-term studies, which makes it impossible to assess the durability of the effects of intermittent fasting and its safety over a period of several months or years. Most of the available studies involved short-term interventions, which limits the ability to assess the impact of IF on disease progression, metabolic complications, or the risk of nutritional deficiencies.

Finally, the review included only publications in English and studies with full-text access (language limitations), which may have led to the omission of relevant data published in other languages or in a format not available in full text. This limitation may affect the representativeness and completeness of the collected data. The literature included in the paper is poor because there are not many scientific reports on the topic discussed in this review, suggesting the need for further research.

Despite these limitations, this review provides an up-to-date, comprehensive, and broad analysis of the available evidence regarding the role of intermittent fasting in rheumatoid arthritis, serving as a foundation for further, more advanced clinical studies.

## Figures and Tables

**Table 1:** Summary of Search Strategy.

Element	Detailed Description
Databases searched	PubMed, Scopus, Web of Science, EBSCO (Medline Complete, CINAHL), Cochrane Library, Google Scholar. The selection of databases reflects the JBI’s recommendations regarding comprehensive evidence mapping and the interdisciplinary nature of the topic (rheumatology, nutrition, immunology, lifestyle medicine).
Search timeframe	The search was conducted from 16 March 2026 to 12 April 2026.
Primary search concepts	“intermittent fasting”, “fasting”, “time-restricted eating”, “periodic calorie restriction”, “caloric restriction”, “rheumatoid arthritis”, “RA”, “autoimmune disease”, “inflammatory arthritis”.
Search strings	Keyword combinations combined with operators AND/OR, np.: (“intermittent fasting” OR “time-restricted eating” OR “periodic calorie restriction”) AND (“rheumatoid arthritis” OR “RA”).
Search fields	Title, abstract, keywords; full text available on Google Scholar.
Filters applied	Language: English; population: humans; availability: full text; no restrictions on publication year.
Inclusion criteria	Observational studies (cross-sectional, cohort, and case–control), experimental studies (randomized and non-randomized), systematic and narrative reviews, publications on intermittent fasting in RA, and in vivo studies using animal models of RA.
Exclusion criteria	Case reports, letters to the editor, commentaries, book chapters, full-text unavailable, languages other than English.
Screening process	Preliminary selection based on titles and abstracts; final selection after reviewing the full texts.
Conflict resolution	Disagreements were resolved through discussion among the research team until full consensus was reached.
Grey literature	Google Scholar was used to identify gray literature and publications not included in traditional databases.
Rationale for database selection	Combining biomedical, multidisciplinary, and complementary databases maximizes the sensitivity of the search and minimizes the risk of overlooking relevant studies.
Search governance	The strategy was developed in accordance with PRISMA ScR, the JBI Manual for Evidence Synthesis, and the principles of search transparency.
Search structure	The search was conducted as a single, integrated search covering all terms; the thematic breakdown took place only during the data selection and extraction phase.

**Table 2:** Characteristics and findings of studies included in this review.

Author, Year	Country	Aim of the Study	Participants	Type of Research	Results and Findings	Fasting Protocol	Overall Assessment
			**Human Clinical and Observational Studies**				
Ben Nessib D. et al., 2022 [9]	Tunisia	An assessment of the positive effects of intermittent fasting during Ramadan on Rheumatoid Arthritis (RA)	35 patients with RA	Retrospective cohort analysis—an observational study	- Intermittent fasting may lead to a rapid improvement in rheumatoid arthritis symptoms - The positive effects of this fasting regimen may last up to 3 months	Ramadan fasting (dawn-to-dusk)	P
Ranjbar M. et al., 2025 [10]	Iran	An assessment of the effects of intermittent fasting in overweight and obese postmenopausal women with RA	44 women	Randomized Controlled Trial	- IF has a beneficial effect on certain outcomes related to patients with RA - IF has no significant effect on inflammation and markers of oxidative stress	16:8 Time-Restricted Eating	P/N
Tavakoli A. et al., 2025 [11]	Iran	An assessment of the effects of intermittent fasting (IF) on antioxidant and inflammatory markers and liver enzymes in postmenopausal, overweight and obese women with RA	44 women with RA	Randomized Controlled Trial	- Intermittent fasting (16:8) has been shown to have a beneficial effect on certain markers of oxidative stress, inflammatory markers, and liver enzymes in postmenopausal, overweight, and obese women with RA - IF may be an effective nonpharmacological intervention in the treatment of RA	16:8 Time-Restricted Eating	P
Ranjbar M. et al., 2024 [12]	Iran	To investigate the effect of IF on quality of life, clinical symptoms, inflammation, and oxidative stress in overweight and obese postmenopausal women with RA	44 patients with RA	Randomized clinical trial	- Intermittent fasting (IF) in postmenopausal women with RA who are overweight or obese leads to a significant improvement in quality of life (QoL) parameters and alleviation of certain clinical symptoms. - The effect of this intervention on systemic markers of inflammation and oxidative stress requires further verification due to inconclusive results.	16:8 Time-Restricted Eating	U
			**Reviews and Meta-Analyses**				
Hansen B. et al., 2025 [21]	Luxembourg	An assessment of the impact of fasting and caloric restriction on rheumatoid arthritis in humans	N/A	Review	- Fasting is a promising adjunctive therapy for RA - The literature on fasting in RA is limited and consists mainly of short-term studies, with limited research on long-term outcomes	Various protocols	U
Hansen B. et al., 2026 [22]	Luxembourg	An assessment of the preventive and therapeutic potential of fasting and nutrition in RA and their possible applications in the context of hormonal fluctuations and life transitions in women	N/A	Review	- Dietary strategies, such as anti-inflammatory and plant-based diets, influence the gut microbiome and may support weight management, improve metabolic health, and reduce oxidative stress—all of which can contribute to better outcomes in RA - These approaches offer promising complementary strategies for improving RA management and enhancing patients’ quality of life	Various protocols	P
Liu R. et al., 2026 [13]	China	To systematically evaluate the overall effects of IF on disease activity and inflammatory markers in patients with rheumatic diseases	N/A	Meta-Analysis	- These findings suggest that intermittent fasting has a positive effect on disease activity, as indicated by both observational studies and RCTs	Various protocols	P
Gunes-Bayir A. et al., 2023 [23]	Turkey	An assessment of the role of diet, including natural products, in the management of rheumatoid arthritis	N/A	Review	- Calorie restriction and intermittent fasting have been shown to benefit patients with RA - Dietary approaches with anti-inflammatory properties may help delay the onset of RA and/or improve symptoms	Various protocols	P
			**Preclinical Animal Studies**				
Cuevas-Martínez R. et al., 2024 [9]	Mexico	To investigate the effects of IF in a murine model of RA	16 male mice	Retrospective cohort analysis	- IF exerts significant anti-inflammatory and immunoregulatory effects in the context of collagen-induced arthritis (CIA), but further research is warranted on the potential benefits of IF in patients with RA.	Intermittent fasting in murine model	U

Table legend: P—positive finding (confirming the benefits of IF); N—negative result (no significant effect); U—mixed/inconclusive finding (requires cautious interpretation in the clinical context).

## Data Availability

No new data were created or analyzed in this study. Data sharing is not applicable to this article.

## Abbreviations

The following abbreviations are used in this manuscript:

RA	Rheumatoid arthritis
IF	intermittent fasting
PCC	Population–Concept–Context
DMARDs	disease-modifying antirheumatic drugs
TRE	time-restricted eating
ADF	alternate-day fasting
JBI	Joanna Briggs Institute
CIA	collagen-induced arthritis
QoL	quality of life
RCTs	randomized controlled trials
P	positive result (confirming the benefits of IF)
N	negative result (no significant changes)
U	mixed/ambiguous result (requires cautious interpretation in the clinical context)
